# The Effects of Bag Style on Muscle Activity of the Trapezius, Erector Spinae and Latissimus Dorsi During Walking in Female University Students

**DOI:** 10.1515/hukin-2015-0005

**Published:** 2015-04-07

**Authors:** Rebecca Hardie, Rachel Haskew, Joel Harris, Gerwyn Hughes

**Affiliations:** 1 Sport, Health and Exercise Subject Group, University of Hertfordshire, UK.

**Keywords:** electromyography, bag style, spinal muscles

## Abstract

Back pain is common in adolescents which has been associated with carrying a bag. However, there is little research examining the effects of bag style in female adolescents. The aim of the study was to investigate the effects of different bag conditions on muscle activity of the trapezius, erector spinae and latissimus dorsi muscles in female university students during walking. Twelve female university students walked on a treadmill for 5 minutes at 1.1 m/s during five conditions; control, 1 strapped rucksack, 2 strapped rucksack, ipsilateral shoulder strap and contralateral shoulder strap, each containing 10% bodyweight. Electromyography for the trapezius, erector spinae and latissimus dorsi was recorded for the last 30 s of each condition. Two-way ANOVA and paired t-tests were used to identify differences between right and left muscles and between bag conditions. Results showed that muscle activity of the left trapezius was significantly higher than the right trapezius during the 1 strap rucksack condition. For the left trapezius, the 2 strapped rucksack and the control condition had significantly lower muscle activity compared to the 1 strapped rucksack and the ipsilateral shoulder strap. For the left erector spinae muscle, there was significantly greater muscle activity when wearing the contralateral shoulder strap compared to the control. For the right erector spinae, significantly lower muscle activity was observed when wearing the 2 strapped rucksack compared to the ipsilateral shoulder strap and contralateral shoulder strap. There were no significant differences in muscle activity of the latissimus dorsi muscles between any of the bag conditions. These findings suggest that a two strapped rucksack should be used when carrying loads to reduce spinal muscle activity which may, in turn, reduce reports of back pain in female adolescents.

## Introduction

Adolescent back pain complaints are increasing with up to 75% experiencing back pain (Jones and Macfarlane, 2005; Skoffer, 2007). Bag carrying has been associated with back pain (Grimmer et al., 2002) with 50% of adolescents with back pain reporting links to bag carriage (Dockrell et al., 2006; Haselgrove et al., 2008; Skoffer, 2007). Back pain due to carrying a bag may occur as a result of increased activity of the back muscles (Elfving et al., 2003) which, in turn, causes muscular fatigue (Ibrahim, 2012; Piscione and Garnet, 2006). Reports of back pain are higher in adolescent females compared to adolescent males (Dockrell et al., 2006; Korovessis et al., 2005), which may be due to females having decreased upper body strength (Haselgrove et al., 2008).

University students commonly carry a bag for more than 30 min daily (Haselgrove et al., 2008) with the average bag load being approximately 10% body weight (BW) (Heuscher et al., 2010). The weight carried has been shown to have little effect on posture or muscle activity (Brackley et al., 2009; Grimmer et al., 2002), whereas the bag type and carrying method have been shown to significantly affect muscle activity and posture (Hong and Xian Li, 2005; Kim et al., 2008; Korovessis et al., 2005; Motmans et al., 2006). Common carrying methods used by adolescent teenagers are a 2 strapped (Dockrell et al., 2006; Motmans et al., 2006) or 1 strapped rucksack (Feingold and Jacobs, 2002), a shoulder bag (bag hangs from one shoulder strap on the same side of the body) (An et al., 2010), a satchel (bag hangs from one shoulder strap across to the opposite side of the body) (Hong and Xian Li, 2005) and a handbag (An et al., 2010).

Asymmetrical bag carrying has been shown to cause an asymmetrical gait (Cottalorda et al., 2004) and decreased stride length (An et al., 2010). Also, previous research has concluded that single strapped bags affect posture by causing, increased cranialthoracic spine rotation (Vacheron et al., 2000), increased shoulder elevation (Korovessis et al., 2004) increased pelvic tilt (Vacheron, 2000), increased trunk lateral flexion, due to weight being on one side of the spine (Pascoe et al., 1997) and increased lumbar flexion and lordosis of the spine (Betanny-Saltikov and Cole, 2012). These postural changes will likely cause an increase in muscle activity of the back muscles responsible for maintaining posture in an attempt to maintain the location of the centre of mass over the base of support during the gait cycle (Grimmer et al., 2002). This increased muscle activity has been linked with increased back pain and injury (Chow et al., 2011). However, previous research examining the effects of single strapped bags on muscle activity presents conflicting findings. For example, Qureshi and Shamus, 2012 reported increased contralateral muscle activity with asymmetrical bag carriage compared to symmetrical bag carriage, whereas Piscione and Garnet (2006) reported no difference in muscle activity between symmetrical and asymmetrical carriage.

Therefore additional research is required to further investigate the link between bag styles and activity of the muscles which maintain posture of the body and may be associated with back pain. Furthermore, there is little research examining muscle activity for a wide range of different bag styles and there is a lack of research in the current literature examining an all-female population, which is the population most at risk of back pain (Dockrell et al., 2006). Therefore, the aim of this study was to investigate the effects of different bag conditions on muscle activity of the trapezius, erector spinae and latissimus dorsi muscles bilaterally in female university students during walking.

## Material and Methods

### Participants

Following institutional ethical approval, a convenience sample of 12 female university students (age = 20.6 ± 1.16 yrs; body height = 1.65 ± 0.04 m; body mass = 69.7 ± 11.3 kg) volunteered to participate in the study. Subjects were free of any lower limb or back injuries at the time of testing and provided written and verbal informed consent before the commencement of the study.

### Procedure

**Figure 1:**
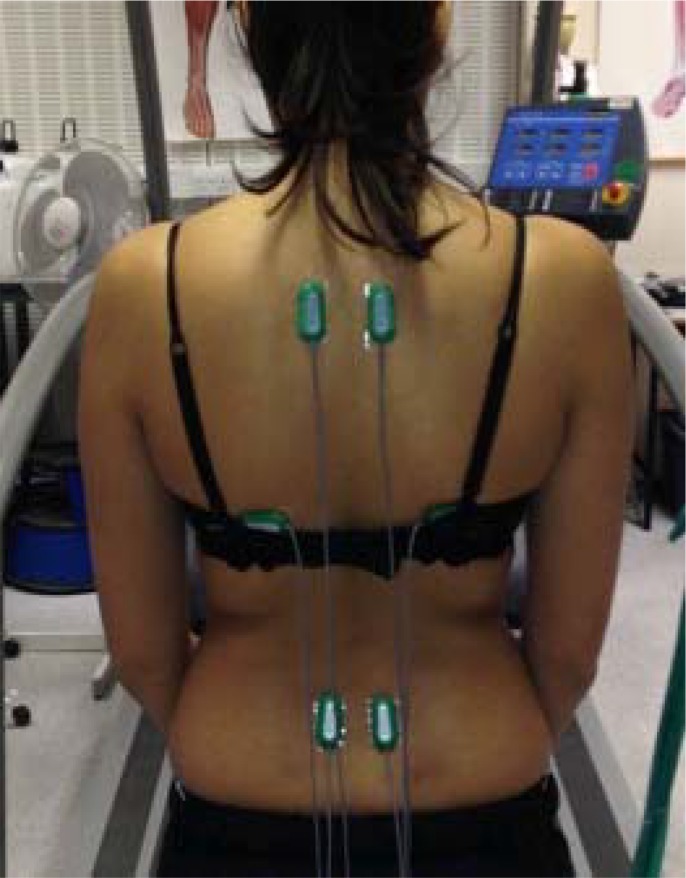
Electrode placement on the trapezius, erector spinae and latissimus dorsi muscles

Subjects attended a single testing session wearing suitable trainers and sport’s clothing. Muscle activity was measured using surface electromyography (EMG) (Biometrics Ltd, Newport, UK) with a sampling rate of 1000 Hz. The EMG was pre-amplified and set at a bandwidth of 20–450 Hz, noise ratio of <5 µV and rejection ratio of 60 Hz (dB>96dB). Surface electrodes were bilaterally fixated to skin parallel to the muscle fibres (using adhesive stickers and tape) to the anatomical locations recommended by SENIAM and Motmans et al. (2006). Integral dry reusable surface electrodes (SX230 Biometrics) with a fixed inter-electrode distance of 20 mm were attached using adhesive pads (T350). The skin of each electrode site was cleaned using alcohol wipes. The trapezius electrode site was located 2 cm lateral to the third thoracic vertebrae, erector spinae was located 2 cm lateral to first lumbar vertebrae and latissimus dorsi was located 2 cm distal to the inferior angle of the scapula (Figure 1). An earth electrode was placed on the wrist.

**Figure 2:**
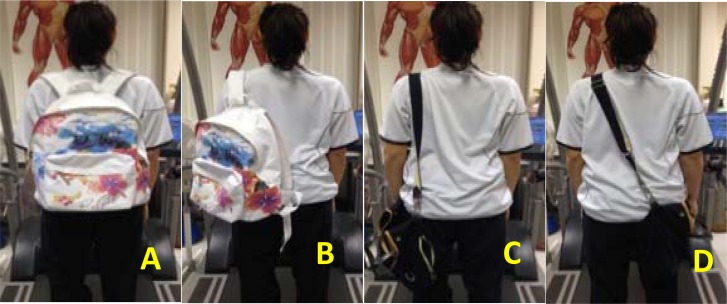
Bag designs and carrying methods. A- 2 strap rucksack, B- 1 strap rucksack, C- Ipsilateral shoulder strap, D- contralateral shoulder strap

The subjects were required to complete a walking task during five bag conditions; 1) a 2 strap rucksack condition, 2) a 1 strap rucksack condition, 3) an ipsilateral shoulder strap condition, 4) a contralateral shoulder strap condition, and 5) a no bag condition (control) (Figure 2). Bags were loaded to be equal to 10% of each subject’s BW. A bag of 10% BW replicates a university student’s load more accurately when using male and female subjects, however, there is no female only guidance on bag weight (Dockrell et al., 2006). The order in which subjects were required to perform the bag conditions was randomised to prevent fatigue and order effects and all single strapped bags were worn on the left shoulder to allow comparison and prevent habitual carrying affecting results. Subjects were instructed to walk on a treadmill at a speed of 1.1 m/s for 5 min facing forwards and adopting their usual walking pattern with the given condition. EMG for all muscles was recorded during the last 30 s of each walking trial. On completion of all bag conditions, manual maximal voluntary contractions (MVC) were conducted for the trapezius, erector spinae and latissimus dorsi. The MVC for the trapezius involved subjects maximally shrugging their shoulders in response to a resistance pushing back down. For the erector spinae, subjects were required to lay prone on the floor and then contract the back muscles to lift feet and arms off the ground to make a dish shape. For the latissimus dorsi, the MVC tests involved the subjects sitting on a bench, then placing both fists either side of their body and lifting themselves off bench whilst extending their legs out in front of the body. All contractions were held for 5 s.

### Statistical Analysis

EMG recordings were analysed using Biometrics’ Datalink DLK900 (version 5.02) software. Raw EMG data for the MVC and both tasks were processed at a five millisecond root mean square (RMS) moving window. EMG data were normalised by dividing the value recorded during the dynamic tasks by the value recorded during MVC task and then multiplying by 100 to express the value as a percentage.

IBM SPSS statistics 21 was used for statistical analysis. A within subjects repeated measures two-way ANOVA was conducted for differences in muscle activity between bag conditions (p<0.05). Further paired t-tests were run to calculate where differences lay between bag conditions and muscles. Since multiple t-tests were carried out, to limit the chance of statistical error due to multiple comparisons a Bonferroni adjustment to the alpha level was made.

## Results

### Trapezius

Muscle activity of the left trapezius was significantly higher than the right trapezius during the 1 strap rucksack condition (t_(11)_ = 3.70, p = 0.004, ɳ^2^ = 0.55). Whilst there was a general trend towards greater muscle activity for the left trapezius than the right across most bag conditions, there were no other significant differences during any of the other bag conditions (Table 1). For the left trapezius, there was a significant effect for the bag condition (F _(4, 8)_ = 7.79, p = 0.007, ɳ_p_^2^ = 0.80), where the 2 strapped rucksack had significantly lower muscle activity compared to the 1 strapped rucksack (t_(11)_ = 6.11, p < 0.001, ɳ^2^ = 0.77) and the ipsilateral shoulder strap (t_(11)_ = 5.31, p < 0.001, ɳ^2^ = 0.72). There was also significantly lower muscle activity during the control condition compared to the 1 strapped rucksack (t_(11)_ = 6.07, p < 0.001, ɳ^2^ = 0.77) and the ipsilateal shoulder strap (t_(11)_ = 5.52, p < 0.001, ɳ^2^ = 0.73). There was no significant effect for the bag condition for the right trapezius (F _(4, 8)_ = 2.49, p = 0.126, ɳ_p_^2^ = 0.56) (Table 1).

### Erector Spinae

There were no significant differences in muscle activity between left and right erector spinae muscles during any of the bag conditions (Table 1). For the left erector spinae muscle, there was a significant effect for the bag condition (F _(4, 8)_ = 5.96, p = 0.016, ɳ_p_^2^ = 0.75), with significantly greater muscle activity when wearing the contralateral shoulder strap compared to the control (t_(11)_ = 3.26, p = 0.008, ɳ^2^ = 0.49). For the right erector spinae, there was a significant effect for the bag condition (F _(4, 8)_ = 11.88, p = 0.002, ɳ_p_^2^ = 0.86), where significantly lower muscle activity was observed when wearing the 2 strapped rucksack compared to the ipsilateral shoulder strap (t_(11)_ = 7.66, p < 0.001, ɳ^2^ = 0.84) and contralateral shoulder strap (t_(11)_ = 5.77, p < 0.001, ɳ^2^ = 0.75) (Table 1).

### Latissimus Dorsi

There were no significant differences in muscle activity between left and right latissimus dorsi muscles during any of the bag conditions. For the muscle activity of both the left (F _(4, 8)_ = 1.08, p = 0.426, ɳ_p_^2^ = 0.35) and right (F _(4, 8)_ = 3.97, p = 0.05, ɳ_p_^2^ = 0.67) latissimus dorsi, there was no significant effect for the bag condition (Table 1).

## Discussion

The aim of the current study was to investigate the effects of different bag conditions on muscle activity of the trapezius, erector spinae and latissimus dorsi muscles bilaterally in female university students during walking. The key findings of the present study are that muscle activity of the trapezius and erector spinae muscles were affected by the bag type, whereas the latissimus dorsi muscle activity was not influenced by the bag style.

For the 1 strapped rucksack, left trapezius muscle activity was significantly greater than right, thus suggesting that if the load does not get distributed bilaterally, there is an increased muscle activity of the trapezius muscle on the shoulder that the bag is worn on. The trapezius muscle is responsible for elevation of the scapula, therefore, this finding may suggest greater muscle activity is due to the subjects trying to maintain the scapula position to ensure the strap of the bag is kept over the shoulder whilst the trunk is laterally flexed so that the centre of mass of the body remains over the base of support during the gait cycle (Grimmer et al., 2002). This is likely to have the effect of increasing the chances of injuries to the shoulder complex as a result of compression and fatigue of the working muscles. Previous research supports this as distributing the load over 2 shoulders has been shown to decrease skin irritation (Holewijn and Meeuwsen, 2000) and upper trapezius pain due to less compression of the subacromial space (Kim and Yoo, 2013).

Single strapped bags have been suggested to cause an increase in cranialthoracic spine rotation (Vacheron et al., 2000), shoulder elevation (Korovessis et al., 2004) and shoulder pain (Korovessis et al., 2005). However, Piscione and Garnet (2006) reported no difference in EMG between symmetrical and asymmetrical carriage. A difference between the current study and Piscione and Garnet (2006) is that the current study kept a constant load across bag conditions (10% BW), which has been found to have minimal physiological effects (Hong and Xian Li, 2005), whereas Piscione and Garnet (2006) used incremented bag weights up to 20% BW which may have caused fatigue and the study does not state that a randomised order was used.

For the left trapezius, muscle activity during the control and the 2 strapped rucksack conditions was significantly less when compared to the 1 strapped rucksack and the ipsilateral shoulder strap conditions. This result suggests that having the bag hang down on the same side of the shoulder over which the strap was located (i.e. 1 strapped rucksack and ipsilateral shoulder strap) increases muscle activity of the trapezius compared to when the bag was either located more symmetrically across the back (i.e. 2 strapped rucksack) or when there was no bag being carried (i.e. control).

There was no significant difference in muscle activity of the trapezius muscles between the control and the 2 strapped rucksack, suggesting that when a load of 10% body weight is distributed posteriorly there is no increase in muscle activity required to maintain posture. This contradicts the majority of research that found bags including a 2 strapped rucksack increased trapezius EMG (Kim et al., 2008), trunk forward lean (Hong and Brueggmann, 2000), the shoulder angle (Korovessis et al., 2004) and head forward posture (Brackley et al., 2009).

For the left erector spinae muscle, there was significantly greater muscle activity when wearing the contralateral shoulder strap compared to the control whereas during the 1 strapped rucksack or ipsilateral shoulder strap, there was no significant difference in left erector spinae muscle activity to the control condition. Since the erector spinae muscles are responsible for extension and lateral flexion of the spine, this finding suggests that the contralateral shoulder strap bag requires greater muscle activity of the left erector spinae to maintain a laterally flexed and extended spine during walking (Qureshi and Shamus, 2012). Motmans et al. (2006) concluded that wearing a single strapped shoulder bag resulted in greater erector spinae activity compared to a control. Differences to the findings of this study may be due to Motmans et al. (2006) measuring EMG whilst the subject stood statically whereas the current study required subjects to walk.

For the right erector spinae, significantly lower muscle activity was observed when wearing the 2 strapped rucksack compared to the ipsilateral shoulder strap and contralateral shoulder strap. This suggests that bilateral bag carriage causes lower right erector spinae activity than unilateral bag carriage, similar to the findings of the trapezius muscle activity. This is supported by research concluding single strapped bags cause increased cranialthoracic spine rotation (Vacheron et al., 2000), increased spinal lateral bend (Pascoe et al., 1997) and hip forces compared to bilateral carriage (Neumann et al., 1992). Satchels have been found to change posture with a load of 10% BW compared to a 2 strapped rucksack that does not alter posture until 15% BW (Hong and Xian Li, 2005). Ramadan and Al-Sheeya (2013) found that a 2 strapped rucksack had increased erector spinae activity compared to a control and modified rucksack, in which weight was distributed equally on the spine by pockets. This suggests that distributing weight equally on the spine, rather than the main distribution being at the bottom of the bag results in decreased spinal activity.

Since no significant difference in muscle activity of the erector spinae was observed between the 1 strapped rucksack and the 2 strapped rucksack but differences were observed between the 2 strapped rucksack and the ipsilateral shoulder strap and the contralateral shoulder strap, this suggests that the torque applied to the body may be different when using longer shoulder straps which may in turn increase muscle activity of the contralateral erector spinae. Due to an increased distance between the weight and the shoulder, there may be greater torque being applied to the ipsilateral side. Therefore, the contralateral spinal muscles need to contract to maintain posture (Grimmer et al., 2002) and decrease spinal deviations (Chow et al., 2011; Vacheron et al., 2002).

There is little research focusing on muscle activity of the latissimus dorsi during carrying different bag styles. The findings of the current study show there were no significant differences in muscle activity between left and right latissimus dorsi muscles during any of the bag conditions and no significant difference in muscle activity between any of the bag conditions for both left and right latissimus dorsi muscles. The latissimus dorsi’s main function is to control extension, adduction and internal rotation of the shoulder joint and is only considered to have limited contribution to movement of the spinal column. Therefore, this finding suggests that there is limited altered demand on the shoulder joint itself due to different bag styles and the activity of the latissimus dorsi is not significantly affected due to bag style.

Since there was no significant difference between the control and 2 strapped rucksack for any muscle analysed, these findings suggest that for 10% BW there is no increased muscle activity when carrying a load via this method. This is supported by research stating no difference in metabolic costs when carrying a 2 strapped rucksack compared to a control (Hong and Xian Li, 2005), suggesting no increase in muscle activity due to the muscles not demanding an increase in oxygen supply (Harms et al., 1997). Previous research has shown there to be no change in hip forces (Neumann et al., 1992) or the gait pattern for this carrying 10% BW using a 2 strapped rucksack (Hong and Xian Li, 2005). However, conflicting findings have been reported showing that 2 strapped rucksacks caused increased trunk forward lean (Chow, 2011), trunk and upper arm muscle recruitment (Matuso et al., 2008), increased muscle activity (Motmans et al., 2006), increased anterior spinal displacement (Vacheron et al., 2000) and a decreased craniocervical angle (Korovessis et al., 2005).

The practical implications of the findings of this study are that female university students should carry loads in a 2 strapped rucksack to cause the least muscle activity which could possibly decrease reports of back pain. Whilst it is acknowledged that students often choose their bag based on fashion and therefore may still choose asymmetrical strapped bags (Mackie et al., 2003), since increased muscle activity has been linked with back pain and injury (Chow et al., 2011), it is recommended that students should be encouraged to carry loads using a 2 strapped rucksack, particularly those who have experienced back pain. This is of particular importance to youth athletes who are likely to be commonly required to carry sporting apparel (i.e. clothing and equipment) required for participation in their chosen sport using bags. Consequently, youth athletes in particular should be encouraged to use 2 strap rucksacks to avoid back pain and spinal asymmetry. Furthermore, future research should investigate the effects of strength training of the back muscles in an attempt to reduce the likelihood of back pain in female adolescents due to carrying bags.

Whilst the current study shows some evidence to support that unilateral bag carriage does alter muscle activity of the trapezius and erector spinae muscles, it should be noted that the current study only looked at posterior muscles, therefore results cannot be applied to muscles of the anterior trunk or lower limb which both could contribute to back pain. Hence future research should examine the anterior trunk muscles and leg muscles to investigate how the bag style affects other areas of the body. Furthermore, an establishment of the association between back pain and muscle activity when carrying bags should be examined by using questionnaires alongside biomechanical assessment.

## Conclusion

This study investigated the effects of different bag conditions on muscle activity of the trapezius, erector spinae and latissimus dorsi muscles bilaterally in female university students during walking. The main findings were that muscle activity of the left trapezius was significantly higher than the right trapezius during the 1 strap rucksack condition. For the left trapezius, the 2 strapped rucksack and the control condition had significantly lower muscle activity compared to the 1 strapped rucksack and the ipsilateral shoulder strap. For the left erector spinae muscle, there was significantly greater muscle activity when wearing the contralateral shoulder strap compared to the control. For the right erector spinae, significantly lower muscle activity was observed when wearing the 2 strapped rucksack compared to the ipsilateral shoulder strap and contralateral shoulder strap. There were no significant differences in muscle activity between the latissimus dorsi muscles between any of the bag conditions. These findings suggest that female university students should carry loads in a 2 strapped rucksack to reduce muscle activity of the trapezius and erector spinae muscles which may potentially reduce back pain. Asymmetrical bag carrying should be avoided due to this causing increased muscle activity. Therefore, it is recommended that students should be encouraged to carry loads using a 2 strapped rucksack, particularly those who have experienced back pain. Future research should focus on investigating the effects of the bag style on anterior and lower limb muscle activity.

## Figures and Tables

**Table 1:** Muscle activity of the Trapezius, Erector spinae and Latissimus dorsi muscles during the different bag conditions (mean ± standard deviation)

	Control (%MVC)	1 strap rucksack (%MVC)	2 strap rucksack (%MVC)	Ipsilateral shoulder strap (%MVC)	Contralateral shoulder strap (%MVC)
Trapezius left	3.57 ± 2.00^1, 2^	6.42 ± 2.83^1, 3, 4^	3.02 ± 1.46^4, 5^	8.31 ± 4.58^2, 5^	4.69 ± 2.07
Trapezius right	4.55 ± 3.66	3.75 ± 2.01^3^	3.68 ± 2.66	5.22 ± 5.97	6.1 ± 4.15
Erector spinae left	3.66 ± 3.21^6^	3.3 ± 3.84	3.46 ± 3.98	3.41 ± 2.44	4.87 ± 3.91^6^
Erector spinae right	2.51 ± 0.94	2.88 ± 0.76	2.32 ± 0.73^7, 8^	3.61 ± 1.19^7^	3.02 ± 0.99^8^
Latissimus dorsi left	4.56 ± 3.74	5.22 ± 3.96	4.49 ± 2.9	4.91 ± 4.13	5.59 ± 3.64
Latissimus dorsi right	3.44 ± 1.91	4.38 ± 2.71	4.02 ± 2.59	5.62 ± 3.21	4.99 ± 3.26

1 – 8: Significant difference
